# Neuralgia

**Published:** 1895-06

**Authors:** Henry Blandy


ARTICLE III.

### NEURALGIA.

HENRY BLANDY, L. D. S., EDIN.

When a dentist speaks of neuralgia, he means a nerve pain of a more extended character than odontalgia, or toothache. When a patient complains of pain of a shooting character in the head, the ear or arm, we say he has neuralgia; we examine his teeth carefully, and we generally find a decayed tooth, or teeth, or stumps, which indicate the locality of the nerve lesion which gives rise to the pain. We remove the decayed tooth and we generally cure the patient.

Some little time ago I had a lady patient who had suffered severely from neuralgia in the head; she had been under treatment by a physician for many months; she had been to Smedley's Hydro. for nine months to get up her general health and tone; she had had a course of massage I suppose on the same principle that muscular rheumatism would be treated, and which is treated effectually in that way—but with no improvement. The racking sleep-destroying pain still continued. She did not believe it could be her teeth, but came to have them examined as a forlorn hope.

There was an upper wisdom tooth decayed, but I diagnosed the lower wisdom as the offender, which was apparently quite sound. This tooth was very sensitive to hot air applied by the hot air syringe, and to cold water. But she declined to allow me to extract it, and decided to have the upper tooth out. It was decayed, and of no great value, so against my advice I took it out. In a week or two she returned; there had been no improvement. Had the upper wisdom tooth been a sound and useful tooth, or been capable of being made in one filling, I should, of course, have refused to extract it. I look on the dentist as the arbitrator between the patient and his impatience. The one is a professional and the other an amateur. The proverb "Every man knows where his own shoe pinches" does not hold good with regard to teeth. On this second visit I was able to get my way, and extracted the lower wisdom. I found the nerve cavity full of pus. We all know how painful a simple gathering on the finger may be when the finger swells, and there is heat and great congestion. The tooth cannot swell, and we get the congestion in the confined pulp chamber, consequently there is compression of the delicate nerve filaments, and we get extreme pain. But the pain may not be localized to the tooth, and we get what we call reflex neuralgia. Neuralgia might also be caused by the deposition of secondary dentine in the pulp chamber.

Neuralgia which may sometimes be relieved by dressing the cavity in some tooth—destroying the pulp, clearing it out and filling it. A young lady came with intense neuralgia, saying she thought she would go mad. She paced up and down the surgery, and demanded almost instant extraction of an upper lateral tooth. This was the only decayed tooth she had, and would have left an unsightly gap, and she would have had to be bothered by a plate for one tooth. I injected morphia in her arm and dressed the cavity—destroyed the nerve and eventually filled the tooth with gold, saving it for appearance and mastication. A gentleman brought his doctor to give him an anæsthetic to have an upper molar out, which I stoutly refused to extract, though under threat that he would go elsewhere, and find a dentist more amenable to reason. But he gave way and I saved that tooth.

Another obscure case of neuralgia is the malposition of teeth. A medical man of 50 years complained of great pains all over his head. He dare not take chloroform or even gas. I believed the trouble to be in the upper right cuspid, under which was a large swelling. I injected cocain and went for the stump. but the instrument slipped and I failed. I went deeper and failed again. Then with my saw edge forceps I trephined till I had a very firm hold, and it took a long and strong pull, but when it came I found I had a large cuspid which had never been erupted. It was lying nearly horizontally in the alveolus. The other case was nearly similar, in a patient of 45, whose cuspid was under sound bicuspids.

A third of malposition, causing great neuralgic pain was that of the wife of a coachman, aged about 45, She had no bad teeth, and apparently a healthy mouth. All her teeth were present except the left upper wisdom. The second molar was decayed in the crown. I carefully tapped this—she winced. On extraction I found the palatal root broken off nearly to the neck. The fracture was not flat and even across, but slightly cupped, evidently not fractured but absorbed, as the roots of temporary teeth are To prove this I immediately extracted the wisdom tooth, which was underlying, and it fitted exactly the second molar palatal root. The same thing sometimes occurs with lower wisdoms, which will burrow under the gum and eat their way in the pulp chamber of the lower second molar. I have the following notes in my hospital case book for last year. Mrs. R., 34, neuralgia 11 years; use of arm gone for 3 or 4 years—3 or 4 days at a time—had not been able to attend to her hair for 5 years. I extracted teeth. Three weeks afterwards she was better and had regained the use of her arm.

Salter gives a case where the right arm was seriously affected—became nearly powerless, and was constantly in a state of aching pain—the patient could hardly grasp or hold anything in her right hand. Facial palsy occurred with dimness of the right eye. A week later she had complete facial paralysis, deafness, and her right arm as above. Upper right wisdom tooth removed. Before patient left the house pain of arm and powerlessness had vanished. Patient quite cured in a fortnight.—*Dental Record.*

